# Combined BRAF^V600E^- and SRC-inhibition induces apoptosis, evokes an immune response and reduces tumor growth in an immunocompetent orthotopic mouse model of anaplastic thyroid cancer

**DOI:** 10.18632/oncotarget.2130

**Published:** 2014-06-26

**Authors:** Pierre Vanden Borre, Viswanath Gunda, David G. McFadden, Peter M. Sadow, Shohreh Varmeh, Maria Bernasconi, Sareh Parangi

**Affiliations:** ^1^ Department of Surgery, Massachusetts General Hospital, Harvard Medical School, Boston, Massachusetts; ^2^ Thyroid Unit, Department of Medicine, Massachusetts General Hospital, Boston, Massachusetts

**Keywords:** Thyroid, BRAF, SRC

## Abstract

Anaplastic (ATC) and refractory papillary thyroid cancer (PTC) lack effective treatments. Inhibition of either oncogenic BRAF or SRC has marked anti-tumor effects in mouse models of thyroid cancer, however, neither drug induces notable apoptosis. Here we report that the SRC-inhibitor dasatinib further sensitizes BRAF^V600E^-positive thyroid cancer cells to the BRAF^V600E^-inhibitor PLX4720. Combined treatment with PLX4720 and dasatinib synergistically inhibited proliferation and reduced migration in PTC and ATC cells. Whereas PLX4720 did not induce robust apoptosis in thyroid cancer cells, combined treatment with dasatinib induced apoptosis in 4 of 6 lines. In an immunocompetent orthotopic mouse model of ATC, combined PLX4720 and dasatinib treatment significantly reduced tumor volume relative to PLX4720 treatment alone. Immune cell infiltration was increased by PLX4720 treatment and this effect was maintained in mice treated with both PLX4720 and dasatinib. Further, combined treatment significantly increased caspase 3 cleavage *in vivo* relative to control or either treatment alone. In conclusion, combined PLX4720 and dasatinib treatment induces apoptosis, increases immune cell infiltration and reduces tumor volume in a preclinical model of ATC, suggesting that the combination of these FDA-approved drugs may have potential for the treatment of patients with ATC or refractory PTC.

## INTRODUCTION

While traditional treatments of papillary thyroid cancer (PTC), including thyroidectomy, thyroid-stimulating hormone (TSH) suppression, and radioactive iodine (RAI) are highly effective at mitigating disease, patients with advanced PTC or undifferentiated (anaplastic) thyroid cancer (ATC) face poor prognosis and need novel therapeutics. Approximately 45% of PTC and dedifferentiated PTC patients have an activating mutation at amino acid 600 of BRAF (BRAF^V600E^) [1, 2]. To improve outcomes for patients with BRAF^V600E^-positive advanced PTC and ATC, efforts have been focused on inhibiting oncogenic BRAF signaling with targeted therapeutics [1, 3-9]. We have previously shown that BRAF^V600E^ plays an important role in the aggressive behavior of thyroid cancer cells *in vitro* and that treatment with the selective BRAF^V600E^-inhibitor PLX4720 results in impressive decreases in tumor volume and metastasis in an orthotopic mouse model of ATC [5-8, 10]. These findings, combined with data from others, led to a phase II clinical trial (NCT01286753) to evaluate the selective BRAF^V600E^-inhibitor vemurafenib in patients with progressive RAI-refractory BRAF^V600E^-positive PTC. Vemurafenib treatment elicited partial responses in a subset of patients. The overall clinical benefit at 6 months was 58% in tyrosine kinase inhibitor (TKI)-naϊve patients and treatment extended progression free survival to 15.6 months [11]. Additionally, in a separate case, a patient with ATC exhibited a response to treatment with vemurafenib [12]. Since complete responses have not been achieved, the results encourage further investigation using more effective combinations to target multiple pathways simultaneously.

While the effectiveness of BRAF^V600E^-inhibition in melanoma has spurred investigation in thyroid cancer, there are important differences between melanoma and thyroid cancer. Nanomolar concentrations of BRAF^V600E^-inhibitors are sufficient to inhibit proliferation in melanoma cell lines whereas micromolar doses are required for a similar effect in thyroid cancer cell lines [6-8, 13, 14]. Furthermore, unlike melanoma cells, thyroid cancer cells do not undergo apoptosis when treated with BRAF^V600E^-inhibitors suggesting the persistence of additional signaling pathways that permit or promote survival [5, 6]. In addition, in melanoma, it appears that oncogenic BRAF-signaling may cloak immune antigens present on the cancer cells and that treating with BRAF^V600E^-inhibitors promotes anti-tumor immune cell infiltration and activity [15-21]. The potential effects of BRAF^V600E^-inhibitors on the immune response have not been investigated in thyroid cancer because all previous studies using human thyroid cancer cells have used immunocompromised mice and novel genetically engineered mice with BRAF tumors rarely show aggressive behavior [22].

In an effort to enhance the efficacy of BRAF^V600E^-inhibition in thyroid cancer, we chose to target additional signaling pathways, chiefly SRC signaling. The *Src* family of kinases consists of 9 members, with *c-Src* being the most prevalent in human tumors and specifically in thyroid cancer cells [23, 24]. The *Src* oncogene, recently identified as a driver of thyroid cancer progression and metastasis, is a compelling therapeutic target as it is an important signaling node that modulates varied downstream signaling including the MAPK, PI3K-AKT, FAK, and STAT3 pathways [23, 25-31]. Activated SRC forms a complex with focal adhesion kinase (FAK) and, together, these two kinases affect cell motility, invasion, proliferation, survival, anchorage-independent growth and drug resistance [24, 32].

A number of SRC-inhibitors have been developed, including dasatinib (BMS-354825), a multikinase inhibitor that is primarily appreciated as a dual SRC- and ABL-family inhibitor [24, 33]. It is FDA-approved for patients with chronic myelogenous and acute lymphoblastic leukemia and is being pursued for patients with solid tumors [23, 27, 34-42]. In spite of many intriguing preclinical findings, SRC-inhibitors have thus far shown limited clinical effectiveness when used as a single agent for solid tumors [30]. More recently, several groups have demonstrated that dasatinib treatment reduces tumor growth and inhibits metastasis in various mouse models of BRAF^WT^ and BRAF^V600E^-positive thyroid cancer [23, 27, 31]. SRC signaling has been implicated in both PTC and ATC with increased expression of pathway members correlating with disease progression, metastatic potential and invasion [23-26, 28, 40, 43-47]. Further, evidence from melanoma, including new data from an unbiased screen for genes conferring resistance to mitogen-activated protein kinase (MAPK) pathway inhibition, suggests that SRC and related *Src* family kinases play a role in resistance to BRAF^V600E^-inhibition [32, 48]. We believe there is potential to use SRC-inhibitors in combination with BRAF^V600E^-inhibitors to treat advanced PTC and ATC.

Our study examines the response of genetically defined murine PTC and ATC cell lines to PLX4720, dasatinib and a combination of these drugs *in vitro*. This approach resonates with the current vision for personalized medicine in which rational therapeutic combinations result in increased efficacy as well as potential for reduced side effects. Additionally, we validate our *in vitro* data in an immunocompetent orthotopic model of aggressive ATC, permitting analysis of the host immune response to the treatment of tumor growth in the thyroid, an aspect of increasing importance given the potential for emerging immunotherapies.

## RESULTS

### Dasatinib effectively inhibits SRC signaling, but not MAPK signaling, in a panel of murine BRAF^V600E^-positive thyroid cancer cell lines

**Figure 1:**
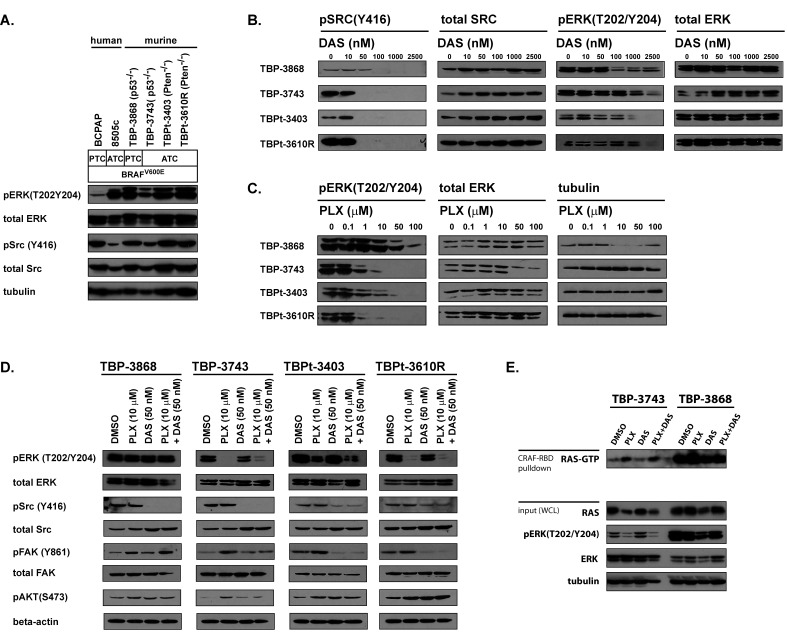
The MAPK and SRC signaling pathways are active and sensitive to PLX4720 and dasatinib in various human and murine BRAF-positive PTC and ATC cell lines A. A panel of human and murine BRAF^V600E^-positive PTC and ATC cell lines were assessed for activated ERK and SRC. Whole cell lysates obtained from BCPAP, 8505c and murine thyroid cancer cell lines deficient for either p53 (TBP-3868 and TBP-3743) or Pten (TBPt-3403 and TBPt-3610R) were probed with pERK(T202/Y204), total ERK, pSRC(Y416), total SRC and tubulin antibodies. B. The murine lines were treated with increasing doses of dasatinib ranging from 0 to 2500 nM for 16 hours and lysed for western blot analysis. C. The murine lines were treated with increasing doses of PLX4720 ranging from 0 to 100 μM where used for 16 hours prior to being lysed for western blot analysis. D. Western blot analysis of cells treated for 16 hours with DMSO, 10 μM PLX4720, 50 nM dasatinib or a combination of 10 μM PLX4720 and 50 nM dasatinib. E. GST-bound CRAF Ras binding domain (RBD) pull down assay to detect GTP-bound RAS in TBP-3743 and TBP-3868 whole cell lysates following treatment with DMSO, 10 μM PLX4720, 50 nM dasatinib or a combination of 10 μM PLX4720 and 50 nM dasatinib for 24 hours.

SRC signaling, as evidenced by phosphorylation at tyrosine 416 (pSRC(Y416)), is active in a panel of human and murine PTC and ATC thyroid cancer cell lines harboring BRAF^V600E^. (Table 1, Fig. 1A). Dasatinib treatment reduces basal SRC signaling in the murine cell lines. Notably, while a complete reduction of pSRC(Y416) is achieved in the murine ATC lines with a dose of 50 nM dasatinib, it is modest in the PTC line, TBP-3868, for which 100 nM dasatinib is required to eliminate pSRC(Y416) (Fig. 1B). Dasatinib treatment does not appreciably effect ERK phosphorylation (pERK(T202/Y204)) at doses lower than 100 nM (Fig. 1B).

**Table 1:** Panel of murine and human thyroid cancer cell lines

species	cell line	type	mutations
murine	TBP-3868	PTC	Braf^V600E/WT^; p53^−/−^
	TBP-3743	ATC	Braf^V600E/WT^; p53^−/−^
	TBPt-3403	ATC	Braf^V600E/WT^; Pten^−/−^
	TBPt-3610R	ATC	Braf^V600E/WT^; Pten^−/−^
human	BCPAP	PTC	BRAF^V600E/WT^; p53^R248G^
	8505c	ATC	BRAF^V600E^/−; p53^D259Y^

(PTC = Papillary Thyroid Cancer, ATC = Anaplastic Thyroid Cancer)

Given that dasatinib does not reduce pERK(T202/Y204) at 50 nM, we treated the murine cell lines with PLX4720. Treatment of the murine ATC cell lines (TBP-3743, TBPt-3403 and TBPt-3610R) with 1 μM of the BRAF^V600E^-inhibitor PLX4720 decreases pERK(T202/Y204), however a more complete reduction is achieved with a dose of 10 μM. Surprisingly, The PTC line, TBP-3868, exhibits a resistance to PLX4720 with pERK(T202/Y204) persisting with doses up to 50 μM (Fig. 1C).

### Combined treatment with PLX4720 and dasatinib inhibits MAPK and SRC signaling and attenuates the PLX4720-induced augmentation of FAK and AKT phosphorylation in a subset of thyroid cancer cell lines

Combined treatment of the ATC cell lines with both 10 μM PLX4720 and 50 nM dasatinib results in a decrease of both pERK(T202/Y204) and pSRC(Y416), whereas only pSRC(Y416) is reduced in the PLX4720-resistant PTC line, TBP-3868 (Fig. 1D).

Notably, phosphorylation of key signaling proteins, FAK and AKT, is observed in lines treated with PLX4720. FAK phosphorylation is induced at tyrosine 861 in all four lines when treated with PLX4720 and combined treatment with both PLX4720 and dasatinib either reduced or eliminated this PLX4720-induced FAK phosphorylation in the ATC lines tested, but not in the PLX4720-resistant line, TBP-3868. PLX4720 induces AKT phosphorylation at serine 473 (pAKT(S473)) in the ATC cell lines. Interestingly, pAKT is not induced in the PLX4720-resistant line TBP-3868, suggesting that the PLX4720-induced increase of pAKT observed in TBP-3743 may be dependent on a decrease in pERK. Combined treatment with dasatinib eliminates PLX4720-induced pAKT in TBP-3473, but not in the PTEN-deficient lines TBPt-3403 and TBPt-3610R.

The PLX4720-insenstive line, TBP-3868, exhibits high basal Ras activity relative to the PLX4720-sensitive line, TBP-3743 (Fig. 1E). BRAF^V600E^-inhibition with PLX4720, activates Ras in the sensitive line TBP-3743. Unlike the PLX4720-mediated activation of FAK and AKT in TBP-3743, the induction of Ras activity is not reduced by combined treatment with dasatinib (Fig. 1E). Interestingly, a shorter film exposure reveals that PLX4720 induces Ras activation in the absence of a reduction of pERK in TBP-3868.

### Dasatinib synergizes with PLX4720 to reduce proliferation in PTC and ATC BRAF^V600E^ cell lines

**Figure 2:**
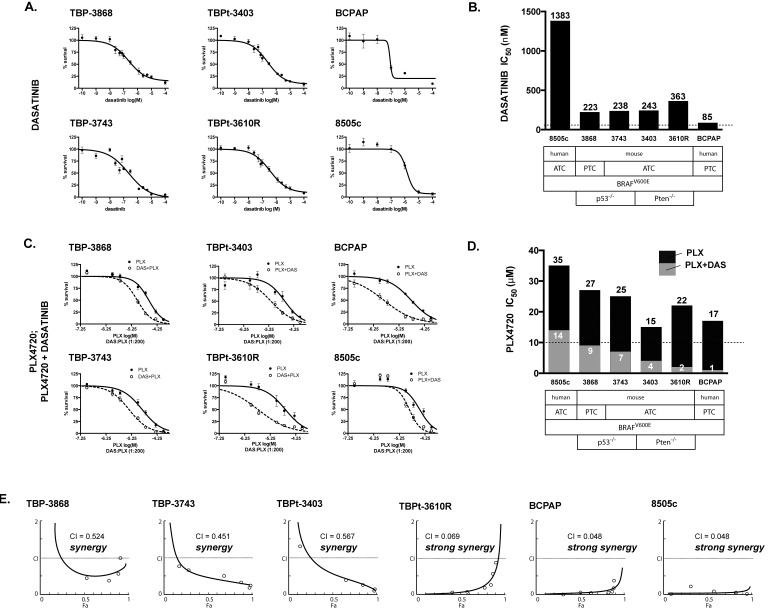
PLX4720 and dasatinib synergistically reduce cellular proliferation in mouse and human PTC and ATC cell lines A. Cellular proliferation curves for the murine TBP-3868, TBP-3743, TBPt-3403 and TBPt-3610R lines and the human BCPAP and 8505c lines treated with a range of doses of dasatinib (0.1 nM to 100 μM). B. Dasatinib IC_50_ values were calculated using GraphPad Prism. C. Proliferation curves for PLX4720 (PLX, 0.1 μM to 100 μM) and PLX4720 plus a fixed ratio dose of dasatinib (PLX+DAS, 200:1). D. PLX4720 IC_50_ values were calculated for cells treated with either PLX4720 alone or both PLX4720 and dasatinib at a 200:1 ratio. E. The interaction between PLX4720 and dasatinib synergistically reduces cell mouse and human thyroid cancer cell proliferation as determined by Chou-Talalay analysis. The combination index (CI), calculated using Compusyn, is less than 1.0 for each line tested indicating synergy.

Dasatinib exhibited a modest anti-proliferative effect on the murine BRAF^V600E^-positive cell lines. The IC_50_ values were determined to be greater than 100 nM, the upper limit of specificity, for each line (TBP-3868 = 223 nM; TBP-3743 = 238 nM; TBPt-3403 = 243 nM; TBPt-3610R = 363 nM). Consistent with previous reports BCPAP exhibited sensitivity (85 nM), while the human ATC line, 8505c, showed resistance to dasatinib (1383 nM) (Fig. 2A-B) [23].

Characteristic of thyroid cancer, the murine and human cell lines exhibited high PLX4720 IC_50_ values (≥ 15 μM). For each cell line, however, co-treatment with a fixed ratio of dasatinib shifted the dose response curve and lowered the PLX4720 IC_50_ value. The IC_50_ values of PLX4720 dropped to less than 10 μM in combination with dasatinib (TBP-3868: 27 μM to 9 μM; TBP-3743: 15 μM to 4 μM; TBPt-3403: 25 μM to 7; TBPt-3610R: 22 μM to 2 μM). Similarly, the IC_50_ value of PLX4720 dropped from 35 μM to 14 μM for 8505c and from 17 μM to 1 μM for BCPAP when combined with dasatinib (Fig. 2C-D).

The combined effect of PLX4720 and dasatinib was formally assessed for synergy by Chou-Talalay analysis [49]. The combination index (CI) for each line was less than 1.0, indicating a synergistic effect between PLX4720 and dasatinib. TBP-3868, which in spite of exhibiting resistance to PLX4720 in terms of the pERK(T202/Y204) inhibition, shows synergy with a CI value of 0.524. A synergistic effect was observed in the ATC lines as well with, CI values of 0.451, 0.567 and 0.069 for TBP-3743, TBPt-3403 and TBPt-3403, respectively. Among the murine lines, TBPt-3610R exhibited the lowest CI value indicating strong synergy (Fig. 2E). Similarly, the human lines exhibited strong synergy between PLX4720 and dasatinib with BCPAP and 8505c both exhibiting CI values less than 0.050 (Fig. 2E).

### PLX4720 has a significant but variable effect on the migration of murine BRAF^V600E^ anaplastic thyroid cancer cell lines whereas dasatinib reduces >90% of the migratory capacity of all cell lines tested

**Figure 3:**
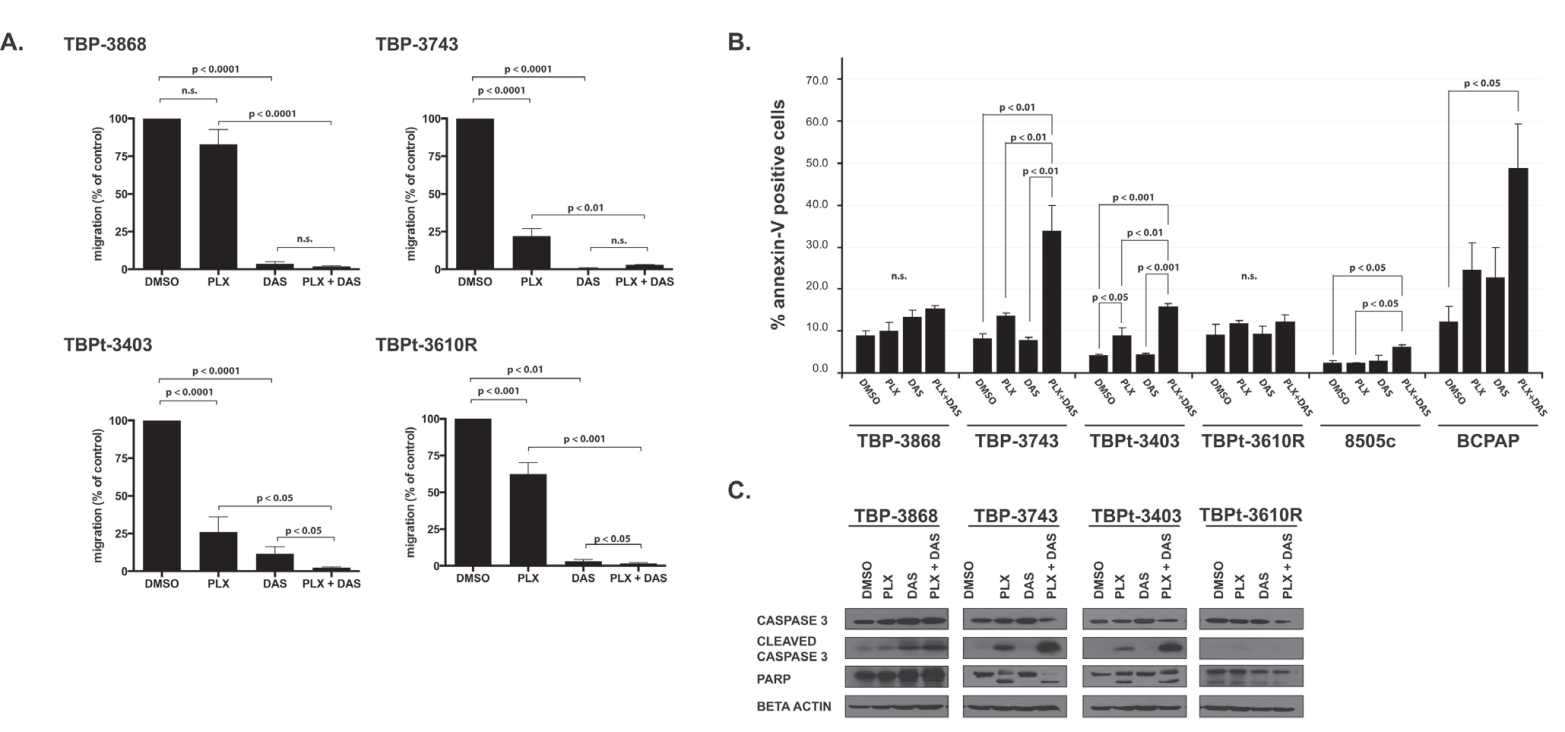
Combined treatment with PLX4720 and dasatinib reduces cellular migration and induces apoptosis in a subset of PTC and ATC cell lines A. PLX4720 has a significant but variable effect on the migration of murine ATC whereas dasatinib strongly reduces the migratory capacity of all cell lines tested. Percent migration relative to DMSO-treated control cells for each murine thyroid cancer cell line was determined following treatment for 16 hours with DMSO, 10 μM PLX4720, 50 nM dasatinib or a combination of 10 μM PLX4720 and 50 nM Dasatinib prior to seeding into transwell migration chambers, under continuous treatment, for migration along a serum gradient for an additional 24 hours. B. To determine percent apoptosis relative to DMSO-treated controls, annexin-V expression was determined by FACS analysis of the cell lines treated with DMSO, 10 μM PLX4720, 50 nM dasatinib, or both 10 μM PLX4720 and 50 nM dasatinib for 18 hours. C. Western blot analysis of whole cell lysates from treated cells. Membranes were probed for Caspase 3 and PARP. Beta actin was probed to verify equal protein loading. Both the migration (conducted in duplicate) and the annexin-V expression experiments were independently performed three times and the means are displayed with SEM. Significant differences are denoted by p values < 0.05 as determined by Student's t-test for the migration assays and by one-way ANOVA followed by Tukey multiple comparison test for the annexin-V analysis.

We previously demonstrated that PLX4720 effectively inhibits the migration of the human ATC line, 8505c [6]. Here we show that the migratory capacity of the murine ATC lines TBP-3743, TBPt-3403 and TBPt-3610R are significantly reduced by treatment with PLX4720. TBPt-3610R is less sensitive to PLX4720 treatment than either TBP-3743 or TBPt-3403. While PLX4720 treatment reduces the migration of TBP-3743 and TBPt-3403 to 22% and 26%, respectively, the migration of TBPt-3610R is reduced to only 63% of DMSO-treated control cells (Fig. 3A). In concordance with the demonstrated resistance to PLX4720-mediated pERK(T202/Y204) inhibition in TBP-3868 cells, PLX4720 treatment does not significantly inhibit the migration of this PTC line. In contrast to the variable anti-migratory responses evoked by PLX4720 treatment, dasatinib treatment strongly inhibits cellular migration in each of the cell lines with the migration of TBP-3868, TBP-3743, TBPt-3403 and TBPt-3610R being reduced to 4%, 1%, 12% and 3%, respectively, of control treated cells. Interestingly, treatment of the PTEN-deficient lines, TBPt-3403 and TBPt-3610R, with a combination of PLX4720 and dasatinib induces a further significant reduction of migration relative to treatment with either agent alone (Fig. 3A).

### Combined treatment of thyroid cancer cell lines with PLX4720 and dasatinib induces apoptosis

Treatment with PLX4720 did not induce a significant difference in the percentage of annexin-V positive cells for any of the lines tested except for the PTEN-null ATC line, TBP-3403, which showed a modest but significant increase to with 8.9±1.8% expressing annexin-V, exhibited a modest but significant increase relative to DMSO-treated cells (p < 0.05) (Fig. 3B). The other murine lines treated with PLX4720 showed no significant apoptosis (Fig. 3B).

Dasatinib treatment of the murine lines did not induce apoptosis, with the percentage of annexin-V positive cells not significantly differing between DMSO- and dasatinib-treated cells (Fig. 3B).

Treatment of two of the ATC lines, TBP-3743 and TBPt-3403, with a combination of dasatanib and PLX4720 induced significantly higher percentages of cells to undergo apoptosis relative to either drug alone. Whereas only 8.3±0.9% of DMSO-treated TBP-3743 cells exhibited annexin-V expression, combined PLX4720 and dasatinib treatment induced 34.0±6.0% of the cells to express annexin-V (p < 0.01) (Fig. 3B). Similarly, a significant increase from 4.2±0.3% to 15.9±.07% is observed in TBPt-3403 (p < 0.001) (Fig. 3B). In contrast, TBP-3868 and TBPt-3610R lines proved refractory to both single agents and the combined treatment, with only 15.3±0.8% and 12.3±1.5% of cells expressing annexin-V following treatment with a combination of PLX4720 and dasatinib (Fig. 3B).

While neither PLX4720 nor dasatinib were effective at inducing apoptosis in human 8505c ATC cells (2.4±0.1%and 3.0±1.2%, respectively, compared to 2.4±0.5% in vehicle treated cells) the combined treatment increased the amount of apoptosis to 6.3±0.3% of the cells (p < 0.05). The human PTC line, BCPAP, has been previously shown to be sensitive to dasatinib and while control cells exhibited relatively high levels of annexin-V positive cells (12.3±3.6%), treatment with PLX4720 or dasatinib increased the percentage to 24.6±6.5% and 22.7±7.2%, respectively. The combination of PLX4720 and dasatinib significantly increased the amount of apoptosis to 48.9±10.5% (p < 0.05).

The flow cytometry findings are supported by western blot analysis of pro-apoptotic proteins following treatment of the murine thyroid cancer lines with PLX4720 and dasatinib. PARP cleavage and Caspase 3 cleavage are observed in both TBP-3743 and TBPt-3403 when these lines are treated with both PLX4720 and dasatinib (Fig. 3C).

### Combined dasatinib and PLX4720 treatment reduces tumor volume in a pre-clinical mouse model of aggressive ATC

**Figure 4:**
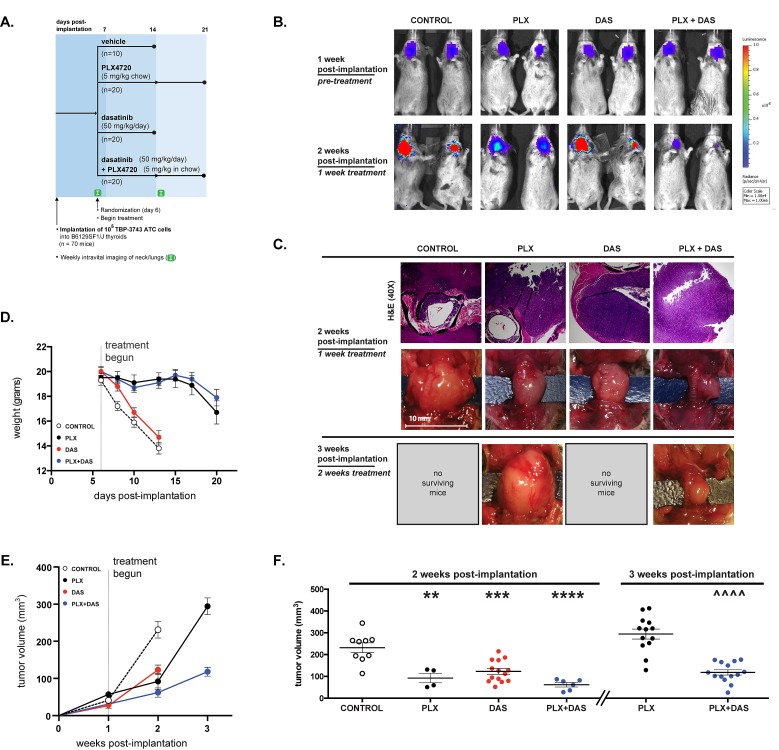
Tumor volume is significantly reduced by combined treatment with both PLX4720 and dasatinib A. 70 B6129SF1/J mice were each orthotopically implanted with 10^5^ TBP-3743 luciferase- and GFP-expressing ATC cells and randomized into four groups 6 days later to receive control chow, PLX4720 chow, 50 mg/kg dasatinib 5 days a week, or both PLX4720 chow and 50 mg/kg dasatinib. Pre-treatment luciferase activity was assessed 6 days post-implantation and again at 14 days post-implantation. Due to loss of weight, control and dasatinib-treated mice were euthanized 14 days post-implantation and PLX4720- and combination-treated mice were euthanized 21 days post-implantation. B. Tumor development in the neck is evident by pre-treatment luficerase activity in B6129SF1/J mice 6 days following the orthotopic implantation of 10^5^ TBP-3743 ATC cells. Mice were imaged again at 13 days post-implantation following one week of treatment with control chow, PLX4720, dasatinib or a combination of the PLX4720 and dasatinib. C. Histologic sections of tumor sections two weeks post-implantation stained with hematoxylin and eosin and gross morphology at two and three weeks post-implantation. D. Mean mouse body weight for each group was determined 6 days post-implantation at the start of treatment. E. Tumor volume was determined by direct measurement of the tumor. Two weeks post-implantation, the mean volume of tumor in control mice was significantly larger than the mean volume of tumors in mice treated with PLX4720, dasatinib, or a combination of the drugs for one week. F. Individual tumor volumes at two and three weeks post-implantation. Mean and SEM with significance determined by one-way ANOVA followed by Tukey multiple comparison test (** p < 0.01, *** p < 0.001, **** p < 0.0001 relative to control at 2 weeks; ^^^^ p < 0.0001 relative to PLX at 3 weeks).

To investigate the efficacy of combined PLX4720 and dasatinib treatment *in vivo* we utilized a newly described immunocompetent model of aggressive thyroid cancer. In this model highly aggressive ATC cells, TBP-3743, transduced with constructs expressing luciferase and green fluorescent protein (GFP) were implanted in the thyroids of B6129SF1/J mice as previously described, which results in rapid tumor progression and local invasive behavior, typically resulting in death of the mouse approximately 14 days post-implantation. This model allows analysis of tumor progression with luciferase imaging and also analysis of immune cell infiltration into the tumor (Fig. 4A) [50]. Six days following implantation, pre-treatment luciferase activity was measured to assess tumor take and progression. Equivalent luciferase activity was detected in the neck region of imaged mice indicating marked and consistent tumor growth (Fig. 4B). Gross examination two weeks post-implantation (one week of treatment) revealed that tumors developed to the left side of the trachea near the site of implantation (Fig. 4C). Fluorescence imaging indicated that the primary tumors were GFP-positive, however luciferase live-imaging, post necropsy GFP-imaging or gross inspection indicated distant metastases to the lung (Fig. 4B-C and *data not shown*). The average body weight of mice from both the control and the dasatinib treated groups was significantly lower than that of mice from either the PLX4720 or combined treatment groups two weeks post-implantation, following one week of treatment (Fig. 4D). Mice treated with PLX4720, dasatinib or a combination exhibited markedly smaller tumors than control mice two weeks post-implantation (Fig. 4C, E, F).

In addition to palpable tumors in the neck region, the mice implanted with TBP-3743 ATC and fed a control diet exhibited poor body condition, a dramatic loss of body weight and were euthanized two weeks post-implantation. These control mice developed large thyroid tumors with a mean volume 23152 mm^3^. At two weeks, mice in the dasatinib-treated group exhibited significantly smaller tumors than the control group (123±28 mm^3^; p < 0.001), however similarly presented with poor body condition, exhibited a dramatic reduction in body weight and were euthanized. Mice fed PLX4720-impregnated chow exhibited tumors with a mean volume of 92±68 mm^3^ two weeks post-implantation, significantly lower than the control group (p < 0.01). Mice treated with the combination of both dasatinib and PLX4720 developed tumors with mean volumes of 62±26 mm^3^, significantly smaller than the control mice (p < 0.0001) (Fig. 4E, F). Though the mean tumor volumes of the PLX4720-treated mice and the mice receiving the combined treatment did not significantly differ from the mean tumor volume of the dasatinib-treated group, mice in these groups maintained body weight and appeared healthy (Fig. 4D).

The PLX4720 and combined treatment groups survived for an additional week before mice of the PLX4720 treatment began to exhibit poor body condition. The average body weight of mice from both groups began to decline in the third week post-implantation and the mice were euthanized. Three weeks post-implantation the mean tumor size of the combined treatment group was 118±26 mm^3^, significantly lower than that the PLX4720-treated group, which was 29450 mm^3^ (p < 0.0001). Notably, the mean tumor size did not significantly change in the combined treatment group from two weeks to three weeks post-implantation.

**Figure 5:**
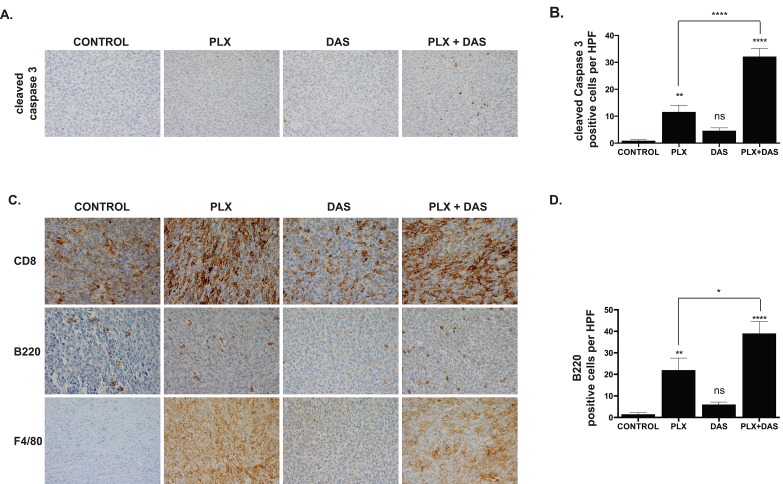
PLX4720 and combined treatment with dasatinib induces caspase 3 cleavage and an immune response characterized by T cell, B cell and macrophage/monocyte infiltration A. Tumor sections obtained from each group two weeks post-implantation were probed for cleaved caspase 3. B. Cells staining positive for cleaved caspase 3 were counted in 10 high powered fields (hpf). Mean and SEM with significance determined by one-way ANOVA followed by Tukey multiple comparison test (** p < 0.005; **** p < 0.0001, n.s. not significant). C. Tumor sections obtained from each group two weeks post-implantation were probed for CD8, B220 and F4/80. D. B220-positive cells were counted in 10 hpf. Mean and SEM with significance determined by one-way ANOVA followed by Tukey multiple comparison test (* p < 0.05, ** p < 0.005; **** p < 0.0001, n.s. not significant).

Caspase 3 cleavage was largely undetectable in tumor sections from control mice suggesting a lack of basal apoptosis (0.9±0.35) (Fig. 5A-B). A modest, but significant, increase in caspase 3 cleavage was observed in PLX4720-treated mice (11.6±2.46; p < 0.01), but not in dasatinib-treated mice (4.6±1.09; p > 0.5) two weeks post-implantation (Fig. 5A-B). Combined treatment resulted in a marked induction of caspase 3 cleavage *in vivo,* significantly increased relative to both control and individually treated mice (32.2±2.94; p < 0.0001) (Fig. 5A-B).

### PLX4720 treatment induces a robust immune response characterized by the infiltration of cytotoxic T cells, B cells and macrophages

The implantation of the thyroid cancer cells initially derived from B6129SF1/J mice into syngeneic mice permits study of immune responses to tumor growth and treatment. Tumor tissue obtained from mice treated with PLX4720 featured a marked increase in the infiltration of CD8+ T cells relative to tumor tissue obtained from the control group (Fig. 5C). Dasatinib treatment did not noticeably induce CD8+ T cell infiltration beyond what was detected in the control group (Fig. 5C). Abundant CD8+ T cell infiltration was observed in tumors obtained from mice receiving combined treatment, however the degree of infiltration did not markedly differ from that observed in mice treated with PLX4720 alone.

Whereas sections obtained from TBP-3743 control tumors two weeks post-implantation are largely devoid of infiltrating B220+ B cells, with 2±0.7 cells per high-powered field (hpf), abundant infiltration of B220+ B cells is observed in tumors treated with PLX4720, with 22±5.6 B220+ cells/hpf (p < 0.001) (Fig. 5C-D). Dasatinib treatment does not induce a significant increase in B cell infiltration relative to control (6±1.1 B220+ cells/hpf), however tumors treated with both PLX4720 and dasatinib feature 39±5.6 B220+ cells/hpf, significantly more than what is observed in tumors obtained from control (p < 0.0001). The increase in B cell infiltration into tumors treated with both PLX4720 and dasatinib is modestly, but significantly, higher than the increase in tumors treated with PLX4720 alone (p < 0.05).

The response of innate immune cells was assessed by probing sections of tumor with a marker of monocytes and macrophages, F4/80. The pattern of infiltration of F4/80+ cells with regard to treatment was similar to the pattern of CD8+ infiltration. Control TBP-3743 tumors were largely devoid of F4/80+ cells whereas tumors from PLX4720-treated mice exhibited marked infiltration. Few F4/80+ cells are observed dasatinib-treated tumors. Tumors obtained from mice receiving combined treatment feature robust infiltration of F4/80+ cells, again, similar to the response observed with PLX4720 treatment alone (Fig. 5C).

## DISCUSSION

Targeted inhibition of oncogenic signaling is emerging as a viable approach for treating thyroid cancer that is refractory to traditional therapies. As in melanoma, the inhibition of oncogenic BRAF has been a major focus of these efforts in thyroid cancer, however the importance of additional pathways has been recognized. Here we investigate the efficacy of the rational combination of FDA-approved inhibitors of oncogenic BRAF and SRC. In addition, our use of a novel immunocompetent orthotopic murine model permits the analysis of the host immune response to thyroid cancer and treatment.

Our data demonstrate that the combined treatment of BRAF mutated thyroid cancer cells with the BRAF^V600E^-inhibitor PLX4720 and the SRC-inhibitor dasatinib results in a synergistic increase in the anti-proliferative and apoptotic responses *in vitro*. Further, combined treatment with these inhibitors results in a robust cytotoxic response and decreased tumor volume in a preclinical model of aggressive BRAF^V600E^-positive ATC. Tumor shrinkage is accompanied by a dramatic host immune response, predominantly driven by PLX4720 treatment and important given the potential for recent innovative immunotherapeutic approaches.

Our lab previously demonstrated that the inhibition of oncogenic BRAF results in dramatic tumor reduction in a model of human ATC and several other groups have shown that dasatinib treatment effectively reduces tumor growth in mice implanted with human BRAF^V600E^-positive thyroid cancer cells [6, 8, 23, 31]. Of the murine lines examined *in vitro*, we selected the most aggressive murine ATC line, TBP-3743, for implantation and investigation in our immunocompetent model. In addition to harboring oncogenic BRAF, TBP-3743 is p53-null and rapidly forms tumors with locally aggressive features [50]. The control animals in the current study recapitulated our previous observations and developed tumors that lead to dramatic weight loss and death two weeks post-implantation. PLX4720 treatment resulted in a significant reduction of tumor size relative to control and extended survival beyond two weeks. Similarly, dasatinib treatment significantly reduced tumor volume however did not extend survival beyond that of control, as the animals treated with dasatinib alone lost weight and became moribund. Dasatinib is metabolized rapidly in humans, with a plasma half-life less than four hours, and it is possible that with appropriate dosing, perhaps by increasing the frequency of doses, the overall efficacy of dasatinib could be augmented [51]. Most importantly, while PLX4720 significantly reduced tumor volume in this model and permitted survival out to three weeks, the combination of PLX4720 and dasatinib resulted in both the greatest reduction in tumor volume and an increase in apoptotic cells.

Both positives and negative aspects are associated with the use of such an aggressive model. A shortcoming of our *in vivo* model is the lack of metastatic progression, putatively due to the rapidity of tumor growth and mortality. Our laboratory has previously demonstrated that PLX4720 treatment effectively blocks distant metastasis in an immunodeficient orthotopic model of ATC utilizing the human line 8505c [6-8]. Similarly, dasatinib inhibited distant metastasis when a human PTC line, BCPAP, was intracardially injected [23]. It is possible that metastases could develop in the model presented here if either fewer cells are implanted or, alternatively, an approach involving intracardial or tail vein injection is used [23, 50, 52]. As executed, while metastases do not form, the model is a stringent test for the efficacy of the treatments, as sizeable tumors develop six days post-implantation when the drugs are first administered [23, 50, 52].

Our experimental design permitted analysis of the immune response to tumorigenesis and treatment. Beyond suppressing tumor growth and inducing apoptosis, the inhibition of oncogenic BRAF evoked an immune-stimulatory effect in mice implanted with TBP-3743. PLX4720 treatment of immunocompetent mice induced the infiltration of CD8+ T cells, B cells and macrophages into the TBP-3743-derived tumor. This observation is consistent with work in melanoma that has demonstrated that BRAF^V600E^-inhibition stimulates the expression of tumor antigens, induces T cell infiltration and enhances the efficacy of infiltrating T cells [15-21]. Dasatinib treatment did not qualitatively induce CD8+ or macrophage infiltration relative to control and the increase in B cell infiltration was not significant, suggesting the increases observed in the tumors treated with both PLX4720 and dasatinib are largely due to PLX4720 treatment. While BRAF^V600E^-inhibition has been previously shown to increase tumor infiltrating lymphocytes, it has also been shown to induce the expression of T cell exhaustion markers including PD-L1, TIM3 and PD1 in melanoma patients [53]. It will be of interest to further characterize the T cell response to BRAF^V600E^-inhibition to determine if combined treatment with immune checkpoint inhibitors will be of benefit in thyroid cancer.

Our *in vivo* findings built on observations *in vitro* which show the combination of PLX4720 and dasatinib to synergistically inhibit proliferation, reduce migration and induce apoptosis. The combined treatment of either murine or human lines with PLX4720 and dasatinib results in a synergistic shifting of the dose response. Synergy is particularly important as it may permit lower doses of PLX4720 to be used, potentially translating to a benefit in the clinic by reducing side effects associated with BRAF^V600E^-inhibition including squamous cell carcinoma [54-56].

Among the cell lines tested, the human ATC line 8505c exhibited resistance to dasatinib, yet synergy was observed with combined treatment. Dasatinib has a wide kinase profile at higher doses and in addition to SRC, dasatinib targets a number of important protein kinases including other SRC family kinases, ABL, KIT, EPH receptors, EGFR, PDGFRA and MAPK proteins including BRAF, CRAF and MEK1 [57]. High doses of dasatinib (100 nM – 2.5 μM) reduced ERK phosphorylation in the murine cell lines suggesting that the anti-proliferative effect of dasatinib treatment may be due, at least in part, to the inhibition of the MAPK pathway. 8505c exhibits the greatest resistance to dasatinib in terms of proliferation (IC_50_ = 1383 nM) and expresses low basal SRC activity relative to the other lines tested, suggesting that the reduced proliferation of 8505c cells treated with high doses of dasatinib (e.g. > 1000 nM) alone may be a result of the direct inhibition of BRAF and/or MEK1 by dasatinib. Further this may also suggest the importance of the higher SRC activity observed in the other lines, all of which showed sensitivity to dasatinib at lower doses.

The inhibition of additional, non-SRC, targets by dasatinib may be particularly relevant following BRAF^V600E^-inhibition that leads to a release of negative feedback and the consequent activation of various signaling proteins by kinome reprogramming [13, 58, 59]. The synergy between dasatinib and PLX4720 could potentially be a result of PLX4720-mediated MAPK-inhibition coupled with dasatinib-mediated SRC-inhibition and dasatinib-mediated inhibition of other protein kinases activated by the loss of MAPK-mediated negative feedback.

TBP-3868 is unique among the lines examined in that ERK phosphorylation is maintained in spite of PLX4720 treatment. BRAF^V600E^ signals as a monomer and independently of upstream Ras activity. In the context of wildtype RAF (BRAF or CRAF) *and* high Ras activity, BRAF^V600E^-inhibition may “paradoxically” enhance MAPK activity [60, 61]. TBP-3868 exhibits high basal Ras activity relative to the PLX4720 sensitive line, TBP-3743 suggesting that the resistance to BRAF^V600E^-inhibition in TBP-3868 may be attributed to Ras-dependent transactivation of RAF dimers. Though we do not observe increased ERK phosphorylation following PLX4720 treatment of TBP-3868 cells, ERK phosphorylation is maintained. Treatment of the PLX4720-sensitive line, TBP-3743, with PLX4720 reduces ERK phosphorylation and induces Ras activity. We did not observe increased pSRC(Y416) in response to PLX4720 treatment, however pFAK(Y861) and pAKT(S473) were elevated, suggesting that BRAF^V600E^-inhibition influences upstream signaling. While increased Ras, FAK and AKT activation following MAPK pathway inhibition is consistent with a release of negative feedback and consequent kinome reprogramming, it is interesting to note that both FAK and Ras are activated by PLX4720 treatment of TBP-3868 in the absence of ERK dephosphorylation. Either, a slight reduction of pERK, imperceptible by western blot analysis, is sufficient for a release of negative feedback or the increased Ras activity is induced by another mechanism. Though SRC may modulate Ras activity via the Ras-GTPase Son-of-Sevenless (SOS), combined treatment with dasatinib does not have an appreciable effect on PLX4720-induced Ras activity in TBP-3743 and only a modest effect on Ras activity in TBP-3868.

PLX4720 is a potent inhibitor of migration, significantly decreasing cellular migration and invasion of human BRAF^V600E^-positive thyroid cancer lines *in vitro* [6]. In spite of this, though significant, the anti-migratory effect of PLX4720 is variable and incomplete in the murine lines. Dasatinib treatment however nearly completely reduced the migratory capacity of all the murine lines examined. Interestingly, while there was no further anti-migratory benefit of combined treatment of the PTEN-intact lines, a modest but significant reduction was observed in the PTEN-null treated with both PLX4720 and dasatinib relative to treatment with dasatinib alone suggesting that the loss of PTEN confers a more migratory phenotype in these thyroid cancer cell lines.

Combined PLX4720 and dasatinib treatment induced apoptosis in a subset of the thyroid cancer lines examined. Beyond modest inductions, neither PLX4720 nor dasatinib triggered robust apoptosis above baseline when used alone but combined treatment with PLX4720 and dasatinib induced significant apoptosis in TBP-3743, TBPt-3403 and in the two human lines tested, 8505c and BCPAP. Though a cytotoxic response to therapy could not be fully predicted based on genotype, differences between the drug response of the PTEN-intact and -null lines did manifest. While apoptosis was induce by combined treatment of the PTEN-intact lines (TBP-3743, 8505c and BCPAP) with the exception of TBP-3868 which is resistant to PLX4720-mediated reduction of pERK, the PTEN-null line TBPt-3610R did not exhibit a cytotoxic response following combined treatment. And though the PTEN-null line TBPt-3403 exhibited a significant increase in apoptosis, it was modest compared to the induction observed in the p53-null line TBP-3743. Dasatinib treatment of TBP-3743 cells reduced pAKT levels, perhaps permitting apoptosis, whereas the high basal pAKT levels in the PTEN-null cells were unaffected by dasatinib treatment, suggesting that the loss of PTEN and the concomitant increase of AKT signaling may be a factor influencing resistance to apoptosis following BRAF^V600E^-inhibition as has been demonstrated in melanoma [62].

In summary, the BRAF^V600E^-inhibitor PLX4720 and the SRC-inhibitor dasatinib synergize to inhibit proliferation, reduce cellular migration and induce apoptosis in a variety of BRAF^V600E^-positive PTC and ATC cell lines. The reduction of tumor growth characterized by increased apoptosis and immune cell infiltration in a preclinical model of ATC further suggest that this therapeutic combination holds promise for patients with aggressive thyroid cancer and may further benefit from additional combination with emerging immunotherapies. Going forward it will be important to determine the effect of combined treatment on metastasized thyroid cancer and over an extended period of time, to elucidate the genetic or epigenetic contributions leading to variable responses to treatment and to further characterize the immune response to treatment. Taken as a whole, these findings now show high potential for clinical impact to patients with BRAF^V600E^-positive anaplastic or refractory papillary thyroid cancer, who currently lack an effective treatment.

## METHODS

### Cell Culture and *in vitro* Drug Treatment

Murine cell lines (TBP-3868, TBP-3743, TBPt-3403 and TBPt-3610R) were previously derived from autochthonous tumors arising in “TBP” mice with thyroid-specific expression of *Braf^V600E^* and thyroid-specific knockout of *p53* (TPOCreER; *Braf^tm1Mmcm/WT^*; *Trp53^tm1Brn/tm1Brn^*) or “TBPt” mice with thyroid-specific expression of *Braf^V600E^* and thyroid-specific knockout of *Pten* (TPOCreER; *Braf^tm1Mmcm/WT^*; *Pten^tm1Hwu/tm1Hwu^*) as described (Table 1) [50] (Mcfadden et al, manuscript submitted). For orthotopic implantation TBP-3743, were previously lentivirally transduced to produce cells expressing both luciferase and green fluorescent protein (GFP) [50]. The murine lines, along with the human ATC line, 8505c (Deutsche Sammlung von Mikrooranismen und Zellkulturen), and the human PTC line, BCPAP (previously provided by Dr. G. Damante of the University of Udine, Italy*)*, were maintained in culture using Dulbecco's modified Eagle's medium (DMEM) supplemented with 10% fetal bovine serum and penicillin/streptomycin.

For *in vitro* treatments, PLX4720 (Plexxikon Inc., Berkeley CA) and dasatinib (TSZChem-BIOTANG, Lexington, MA) were each dissolved in dimethyl sulfoxide (DMSO) to respectively yield 10 mM and 5 mM stock solutions. Cells were treated with either 0.1% dimethyl sulfoxide (DMSO), PLX4720 at concentrations ranging from 0.1 μM to 100 μM, 50nM dasatinib, or a combination of both 10 μM PLX4720 and 50 nM dasatinib overnight, unless otherwise specified.

### Western Blot Analysis

Whole cell lysates (WCLs) were probed with antibodies against phosphor-SRC(Y416), SRC, phospho-FAK(861), FAK, ERK, phospo-ERK(T202/Y404), AKT, phospho-AKT(S473), PTEN, Caspase 3, PARP and tubulin (Cell Signaling Technologies, Beverly, MA) following separation by SDS-PAGE and transfer to a nitrocellulose membrane.

### Ras activation assay

GTP-bound Ras was detected in WCLs obtained from TBP-3743 and TBP-3868 cells treated as above with DMSO, 10 μM PLX4720, 50 nM dasatinib or a combination of both PLX4720 and dasatinib for 24 hours. We used the active Ras detection kit (Cell Signaling Technologies, Beverly, MA) and followed the manufacturer's instructions. In brief, GST-bound CRAF (RAF1) Ras binding domain (RBD) was incubated with 500 μg WCLs. Following sample preparation, input whole cell lysates and pull down samples were separated by SDS-PAGE, transferred to a nitrocellulose membrane and probed for with antibodies against RAS, pERK(T202/Y204), ERK and tubulin.

### Proliferation Assay and Chou-Talalay Synergy Analysis

Cellular proliferation was assessed with CellTiter Aqueous One solution (Promega, Madison, WI). Murine cell lines were plated in triplicate in 96 well plates with 2500 cells per well and treated with varying doses of dasatinib, PLX4720 or a combination for 72 hours. Each experiment was conducted in triplicate and independently repeated three times. Prism (GraphPad Software, San Diego, CA) was used to calculate nonlinear fit of growth curves and determination of IC_50_. Synergy was formally assessed by Chou-Talalay analysis and the combination index (CI) was calculated using Calcusyn (BioSoft, Cambridge, UK).

### Annexin-V Apoptosis Analysis

Cultured cells were collected, washed in PBS, incubated with Annexin-V Alexa Fluor 647 conjugate in Annexin-V binding buffer (Invitrogen, Life Technologies, Grand Island NY) and stained with propidium iodide (Sigma, St. Louis, MO) for analysis on a BD Sorp 8 Laser LSR II (BD Biosciences, San Jose, CA). Flow cytometry data was analyzed using FlowJo (Tree Star Inc., Ashland, OR). Experiments were repeated independently three times and statistical comparison was made with one-way analysis of variance (ANOVA) followed by the Tukey post hoc multiple comparison test. Statistical significance was determined by p-values < 0.05.

### Migration Assay

Migration assays were performed using 2.5x10^5^ cells per well. Cells were treated in DMEM containing 0.1% FCS for 18 hours prior to seeding into 8 μm transwell inserts containing DMEM with 0.1% FCS in the upper well and DMEM with 5% FCS in the lower well. Cells were incubated at 37°C and 5% CO_2_ for 24 hours prior to methanol fixation, Giemsa staining and counting. Three independent experiments were conducted in duplicate and results were analyzed with Student's t-test following normalization of the DMSO-treated samples. p-values < 0.05 were considered significant.

### Orthotopic Implantation and *in vivo* Drug Treatment

All animal work was done at Massachusetts General Hospital (Harvard Medical School) in accordance with federal, local, and institutional guidelines. TBP-3743 cells expressing luciferase and GFP were implanted in the thyroids of B6129SF1/J syngeneic mice as previously described [50, 63]. In brief, thyroids of age-matched female B6129SF1/J mice were unilaterally implanted with 10^5^ TBP-3743-lucGFP cells suspended in 10 l serum-free DMEM. The right thyroid was not manipulated and was used as an internal control. Mice were randomized into four groups 6 days post-implantation and placed on respective treatment regimens. The control group received a control diet (Research Diets, Inc.) (n=9), the PLX4720-treated group received PLX4720-impregnated chow (417 mg/kg, Research Diets, Inc.) *ad libitum* (n=20), the dasatinib-treated mice received control diet and were orally gavaged with 50 mg/kg dasatinib (Toronto Research Chemicals, Toronto, Ontario, Canada) in 80 mM sodium citrate five days per week (n=20), and the combined treatment group received both PLX4720-impregnated chow *ad libitum* and were orally gavaged with 50 mg/kg dasatinib five days per week (n=20). Mice were weighed every other day. Mice were sacrificed and tumor volume was calculated as (π/6) × length × width × height [64]. Prism (GraphPad Software, San Diego, CA) was utilized for statistical analysis of tumor volumes. Statistical difference was determined with ANOVA followed by the Tukey post hoc multiple comparison test and p-values < 0.05 were considered significant.

### Bioluminescence Imaging

Mice were anesthetized with CO_2_ and injected with luciferin for the detection of luciferase activity using an IVIS Imaging System 100 (Perkin Elmer, Waltham, MA) one week post-implantation prior to treatment and two weeks post-implantation after one week of treatment.

### Histological and Immunohistochemical Analysis

Tissue specimens were fixed with 10% buffered formalin phosphate and embedded in paraffin blocks. Hematoxylin and eosin stained sections were evaluated by an endocrine pathologist (P.S.) and processed for immunohistochemical analysis (IHC) as previously described [8]. IHC was performed using CD8 (Leica Microsystems Inc., Buffalo Grove, IL), B220 (BD Pharmingen, San Jose, CA), F4/80 (AbD Serotec, Raleigh, NC) antibodies. Stained and IHC tissue sections were photographed using an Olympus BX41 microscope and the Olympus Q COLOR 5 photo camera (Olympus Corp., Lake Success, NY). Caspase 3 cleavage was quantified in tumor sections by manually counting positive cells in ten 400X fields for each sample.
